# Role of the retinoid X receptor–peroxisome proliferator-activated receptor-γ axis in adolescent attention-deficit hyperactivity disorder

**DOI:** 10.1007/s00406-025-02178-7

**Published:** 2025-12-19

**Authors:** Ju-Wei Hsu, Li-Chi Chen, Ya-Mei Bai, Shih-Jen Tsai, Mu-Hong Chen

**Affiliations:** 1 Department of Psychiatry, Taipei Veterans General Hospital, No. 201, Shih-Pai Road, Sec. 2, Taipei, 11217 Taiwan; 2 Department of Psychiatry, College of Medicine, National Yang Ming Chiao Tung University, Taipei, Taiwan; 3 Department of Psychiatry, General Cheng Hsin Hospital, Taipei, Taiwan; 4 Institute of Brain Science, National Yang Ming Chiao Tung University, Taipei, Taiwan

**Keywords:** Adolescents, ADHD, Retinoid x receptor-α, RXR-PPAR-γ axis, Working memory, Inhibitory control, Enzyme-linked immunosorbent assay

## Abstract

**Background:**

Studies have reported the retinoid X receptor (RXR)–peroxisome proliferator-activated receptor-γ (PPAR-γ) axis, a heterodimeric nuclear receptor complex regulating synaptic plasticity and neuroinflammation, in neurodevelopment, with emerging evidence suggesting its disruption contributes to cognitive impairments akin to those in attention deficit hyperactivity disorder (ADHD).

**Methods:**

This study included 104 adolescents with ADHD and 87 age-matched neurotypical adolescents. All participants completed working memory and go/no-go tasks. Clinical symptoms were assessed using the Swanson, Nolan, and Pelham IV scale and the Child Behavior Checklist Dysregulation Profile. Fasting serum levels of RXR-α, PPAR-γ, and PPAR-γ coactivator 1α were quantified via enzyme-linked immunosorbent assay.

**Results:**

Generalized linear models adjusted for demographic characteristics, ADHD medications, and clinical symptoms revealed that adolescents with ADHD had reduced RXR-α levels (*p* = 0.001; Cohen’s d = 0.47) compared with neurotypical adolescents. No significant between-group difference was noted in the level of PPAR-γ or PPAR-γ coactivator A. Furthermore, RXR-α levels negatively associated with the mean reaction time in the go/no-go task (β = -0.001; Wald χ2 = 0.475; *p* = 0.029).

**Conclusion:**

To the best of our knowledge, this study is the first to demonstrate reduced peripheral RXR-α levels in human adolescents with ADHD, independent of medication status and symptom severity, extending preclinical retinoid signaling evidence. Further investigation is required to elucidate the neuromechanisms linking ADHD to the RXR–PPAR-γ axis.

## Introduction

Attention-deficit hyperactivity disorder (ADHD) is the most prevalent neurodevelopmental disorder and typically emerges in childhood or early adolescence. It is characterized by developmentally inappropriate levels of inattention, hyperactivity, and impulsivity that substantially impair daily functioning. A systematic review estimated that the global community prevalence of ADHD ranges from 2% to 7%, with an average of approximately 5% [1]. Although ADHD is highly heritable, its precise etiology remains unclear. Current evidence suggests that a complex interplay between genetic predispositions and environmental factors contributes to a broad spectrum of neurobiological vulnerability underlying the disorder [1, 2].

Studies have demonstrated the crucial role of the retinoid X receptor (RXR)–peroxisome proliferator-activated receptor-γ (PPAR-γ) axis in neurodevelopment and have suggested that disruption of this axis leads to psychopathologies such as cognitive dysfunction and emotional dysregulation [3]. The RXR–PPAR-γ axis comprises three major components: RXR, PPAR-γ, and PPAR-γ coactivator A (co-A). The ligand-activated transcription factor PPAR-γ is involved in lipid homeostasis, glucose metabolism, adipogenesis, and anti-inflammatory responses [4]. PPAR-γ also regulates neuroprotection and neuroinflammation in the central nervous system [4]. RXR belongs to the nuclear receptor superfamily. Its most well-characterized isoform is RXR-α, which serves as an obligatory heterodimeric partner for PPAR-γ and several other nuclear receptors [5]. The transcriptional coactivator PPAR-γ co-A is essential for mitochondrial biogenesis, energy metabolism, and neural plasticity [6]. It enhances the transcriptional activity of the RXR–PPAR-γ complex, which is thought to modulate the function of major neurotransmitter systems, including cholinergic, glutamatergic, dopaminergic, and GABAergic pathways [6].

Researchers have highlighted the potential role of retinoic acid, which binds to the retinoic acid receptor (RAR), in the pathomechanisms underlying ADHD [7]. A study involving 82 children with ADHD and 106 neurotypical children revealed relatively low retinol levels in children with ADHD and found an association between lower retinol levels and more severe ADHD symptoms, as measured using the Swanson, Nolan, and Pelham IV (SNAP-IV) scale [7]. In animal work, hippocampal retinoic acid levels tracked memory performance in aged rats, consistent with a role for RAR/RXR-mediated transcription in cognitive function [8]. Beyond RAR/RXR, PPAR pathways also appear relevant: in mice, pharmacological activation of PPAR-α facilitated fear-extinction learning and improved emotional regulation, a core symptom of ADHD, whereas antagonism blocked these effects [9]. Genetic disruption of the PPAR-γ co-A produced hyperactivity and heightened emotional reactivity [10]. Akhondzadeh et al. demonstrated that in children with ADHD, *Passiflora incarnata*—which contains quercetin, a known enhancer of PPAR-γ co-A levels—exerted attention-improving effects similar to those of methylphenidate [11]. However, no human studies have assayed the full RXR-PPAR-γ axis in adolescents with ADHD, despite prefrontal cortex developmental links [3–6].

The present study investigated the role of the RXR-PPAR-γ axis in adolescents with ADHD. Specifically, we measured the levels of RXR-α, PPAR-γ, and PPAR-γ co-A and explored their associations with ADHD-related cognitive dysfunction, measured in terms of working memory and inhibitory control. We hypothesized that adolescents with ADHD have lower levels of RXR-α, PPAR-γ, and PPAR-γ co-A than do neurotypical adolescents and that disruption of the RXR–PPAR-γ axis is associated with ADHD-related cognitive impairment.

## Methods

### Participants

In all, we enrolled 104 adolescents aged 12–17 with ADHD given by experienced clinical child and adolescent psychiatrists (JWH, LCC, and MHC) based on the criteria of the Diagnostic and Statistical Manual of Mental Disorders, Fifth Edition (DSM-5), using the Kiddie-Schedule for Affective Disorders and Schizophrenia for School-Age Epidemiologic Version [12]. The exclusion criteria included other major psychiatric disorders (schizophrenia, bipolar disorder, major depressive disorder, delusional disorder, and obsessive-compulsive disorder), as well as intellectual disability, eating disorders, autism spectrum disorder, alcohol or substance use disorders, organic mental disorders, major autoimmune diseases, severe cerebrovascular disease, epilepsy, pregnancy or breastfeeding, and unstable physical illnesses in the present study. All adolescents with ADHD were recruited from the psychiatric outpatient clinics of our hospitals. Among adolescents with ADHD, 54 (51.9%) received the regular ADHD medication treatment with the optimal dose (0.8 ~ 1.2 mg/kg); 50 (48.1%) were medication-naïve. In addition, we also enrolled 87 age-matched neurotypical adolescents, who had no DSM-5 diagnoses based on the formal diagnostic interview and no major physical diseases, as a reference group. Neurotypical adolescents were recruited from the community via the Institutional Review Board-approved advertisement. Mean ages were 14.03 ± 1.83 and 14.22 ± 1.32 years, respectively, in the ADHD and neurotypical groups. Male predominance was noted in the ADHD group (70.2%). The study was powered at 80% to detect a medium effect (d = 0.50) at α = 0.05 using G*Power. Other clinical variables, such as sex, body mass index (BMI), and medications, were included as covariates in the analyses. All participants completed two cognitive tasks, namely the 2-back working memory task and go/no-go task. The 2-back working memory task comprised two runs of eight 20-trial blocks (320 trials total). Within each 2-back block, 6/20 trials (30%) were targets—defined as the current digit matching the one presented two trials earlier (e.g., 27–42–27)—and 14/20 (70%) were non-targets; sequences were pseudorandomized with a minimum of two non-targets between targets. Participants pressed a button to targets and withheld responses to non-targets. A 10-trial practice block was administered, requiring ≥ 80% accuracy to proceed. For reaction-time analyses, anticipatory responses (< 150 ms) and very slow responses (> 2,000 ms) were excluded. The task required continuous monitoring and updating of information in working memory, and responses were recorded based on both accuracy and reaction time to the repeated stimuli. Participants completed two runs of a symbol go/no-go task. On each trial, a single character appeared (go/target = “×”; no-go/non-target = “+”). Stimuli were presented for 300 ms, followed by a 1,000 ms response window and a jittered inter-trial interval (mean = 1,000 ms; range = 700–1,300 ms). Each run comprised 240 trials (480 total), with 75% go (180 per run; 360 total) and 25% no-go (60 per run; 120 total). Trial order was pseudorandomized with no more than three consecutive no-go trials. Participants pressed a key for go targets and withheld responses to no-go non-targets. For reaction-time analyses, anticipatory responses (< 150 ms) and very slow responses (> 2,000 ms) were excluded. Performance was evaluated based on errors, mean reaction time (ms), and standard deviation (s.d.) of reaction times for both tasks. All adolescents with ADHD who received the medication treatment maintained their medications during the cognitive assessment. Two cognitive tasks were commonly used in our previous studies [13, 14]. The parents of all participants fulfilled the SNAP-IV and Child Behavior Checklist Dysregulation Profile (CBCL-DP) [15, 16]. This study was approved by the Institutional Review Board of Taipei Veterans General Hospital (approval number: 2020-07-010 A) and conducted in accordance with the Declaration of Helsinki. All procedures adhered to the ethical standards of the relevant national and institutional committees on human research, as well as the 1975 Helsinki Declaration and its 2008 revision. Written informed consent was obtained from all participants and also from their parents.

### Measurement of RXR-α, PPAR-γ, and PPAR-γ Co-A levels

After an overnight fast (≥ 8 h), blood was collected between 09:00 and 12:00 into serum-separator tubes, allowed to clot for 30 min, and the resulting serum was stored at − 80 °C until analysis. The human PPAR-γ enzyme-linked immunosorbent assay (ELISA) kit, human PPARgC1a ELISA kit, and human RXRa ELISA kit were used to assess the levels of PPAR-γ, PPAR-γ co-A, and RXR-α, respectively. All experiments were conducted in accordance with the vendor’s instructions. Standard curves were prepared across the vendor-specified range and fit with a four-parameter logistic model; curves were accepted when back-calculated standard concentrations were within ± 15% of nominal and residuals showed no systematic trend. Values below the lower limit of quantification (LLOQ) were treated as left-censored and replaced with LLOQ/√2 for descriptive analyses; values above the upper limit of quantification (ULOQ) were re-assayed after appropriate dilution. A linear regression R² value of ≥ 0.95 was considered indicative of a reliable standard curve. Final absorbance values for each sample were measured at 450 nm using an ELISA plate reader (Bio-Tek Power Wave Xs) and analyzed with Bio-Tek’s KC Junior software (Winooski, VT, USA).

### Statistical analysis

The χ^2^ test was employed for categorical variables and the analysis of variance F-test for continuous variables in between-group comparisons. We employed generalized linear models (GLMs) with a gamma log link to measure the levels of RXR-α, PPAR-γ, and PPAR-γ co-A between the ADHD and neurotypical groups after adjusting for age (continuous), sex (categorical), BMI (continuous), ADHD medications (categorical), total SNAP-IV scores (continuous), and total CBCL-DP scores (continuous). The levels of PPAR-γ, PGC-1α, RXR-α were right-skewed, with variability increasing with the mean—features well matched to a gamma variance function with a log link. The standard GLM checks were performed, including goodness-of-fit (Bayesian information criterion), residual analysis (residual-vs-fitted and Q–Q plots), and the outlier check (deviance residual). No outliers were identified (|deviance residual|>3). In order to elucidate whether ADHD medications may affect the significant findings from the above GLMs, the additional GLMs with a gamma log link were performed among three groups, namely drug-naïve adolescents with ADHD, adolescents with ADHD with medications, and neurotypical adolescents, after adjusting for the demographic data and clinical symptoms. Finally, GLMs with a gamma log link and with adjustment of age, sex, BMI, ADHD medications, SNAP-IV scores, and CBCL-DP scores were used to examine associations between the levels of RXR-α, PPAR-γ, and PPAR-γ co-A and cognitive function parameters. A two-tailed P-value of less than 0.05 was considered statistically significant. Data management and statistical analyses were performed in IBM SPSS Statistics, version 30 (IBM Corp., Armonk, NY, USA).

### Data availability

De-identified individual participant data (serum levels, cognitive scores) are available upon verified request to the corresponding author for 3 years post-publication, per Taipei Veterans General Hospital Institutional Review Board policy, excluding raw ELISAs for privacy. However, they are not publicly accessible due to ethical regulations governing clinical trials in Taiwan.

## Results

A total of 104 adolescents with ADHD and 87 age-matched neurotypical adolescents were included in the present study (Table 1). The ADHD group had a higher proportion of males (χ² = 6.843, *p* = 0.011) and a significantly greater mean BMI (F = 6.455, *p* = 0.012) than the neurotypical group (Table 1). Compared with neurotypical adolescents, adolescents with ADHD demonstrated significantly higher scores on the parent-reported SNAP-IV (F = 173.957, *p* < 0.001) and CBCL-DP (F = 172.395, *p* < 0.001) (Table 1). Adolescents with ADHD performed worse in both the working memory task and the go/no-go task than did neurotypical adolescents (Table 1).

**Table 1:** Demographic and clinical characteristics between groups

	Adolescents with ADHD (*n* = 104)	Neurotypical adolescents (*n* = 87)	F/χ²	*p*-value
Age (years, mean, SD)	14.03 (1.83)	14.22 (1.32)	0.649	0.422
Sex (n, %)			6.843	0.011
Female	31 (29.8)	42 (48.3)		
Male	73 (70.2)	45 (51.7)		
ADHD medications (n, %)				
Medication-naïve	50 (48.1)			
With medications	54 (51.9)			
BMI (mean, SD)	22.32 (5.75)	20.51 (3.66)	6.455	0.012
Clinical symptoms				
SNAP-4 (mean, SD)	36.31 (15.00)	12.24 (8.70)	173.957	< 0.001
CBCL-DP (mean, SD)	29.26 (13.91)	7.44 (7.36)	172.395	< 0.001
Cognitive function				
Working memory task				
Errors (mean, SD)	1.61 (7.11)	0.44 (0.82)	2.322	0.129
Reaction time (ms, mean, SD)	876.21 (200.93)	786.53 (173.07)	10.693	0.001
s.d. of reaction times (ms, mean, SD)	271.86 (112.97)	221.23 (90.54)	11.363	0.001
Go/no-go task				
Errors (mean, SD)	1.33 (2.08)	0.36 (0.66)	17.397	< 0.001
Reaction time (ms, mean, SD)	464.62 (83.52)	457.75 (80.00)	0.333	0.565
s.d. of reaction times (ms, mean, SD)	96.81 (36.81)	71.77 (17.86)	33.612	< 0.001

ADHD: attention deficit hyperactivity disorder; BMI: body mass index; CBCL-DP: Childbehavior checklist dysregulation profile; SD/s.d.: standard deviation; SNAP-4: the Swanson,Nolan, and Pelham Rating Scale-IV

**Fig. 1:**
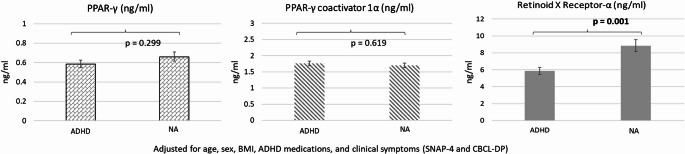
Estimated levels using the GLM models after adjusting for age, sex, BMI, ADHD medications, SNAP-4 scores, and CBCL-DP scores. ADHD: attention deficit hyperactivity disorder; BMI: body mass index; CBCL-DP: Child behavior checklist dysregulation profile; GLM: generalized linear model; NA: neurotypical adolescents; PPAR-γ: Peroxisome proliferator activated receptor-γ; SNAP-4: the Swanson, Nolan, and Pelham Rating Scale-IV. GLM-predicted means ± standard errors were back-transformed from the log scale to the original concentration units to aid interpretation

**Fig. 2:**
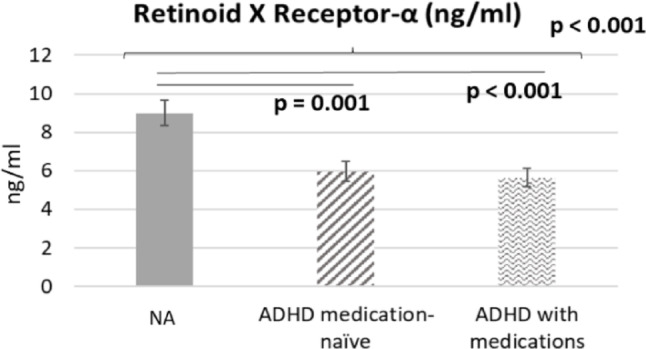
Estimated levels for retinoid X receptor-α among patients with ADHD with and without medications compared with the control group using the GLM models after adjusting for age, sex, BMI, SNAP-4 scores, and CBCL-DP scores. ADHD: attention deficit hyperactivity disorder; BMI: body mass index; CBCL-DP: Child behavior checklist dysregulation profile; GLM: generalized linear model; NA: neurotypical adolescents; PPAR-γ: Peroxisome proliferator activated receptor-γ; SNAP-4: the Swanson, Nolan, and Pelham Rating Scale-IV. GLM-predicted means ± standard errors were back-transformed from the log scale to the original concentration units to aid interpretation

GLMs with adjustment of demographic data, ADHD medications, and clinical symptoms demonstrated that adolescents with ADHD had reduced RXR-α levels (β = -0.411; stand error [SE] = 0.126; exp(B) = 0.663; 95% confidence interval [CI] 0.518–0.848; *p* = 0.001; Cohen’s d = 0.47) compared with neurotypical adolescents (Fig. 1). Levels of PPAR-γ (*p* = 0.299) and PPAR-γ co-A (*p* = 0.619) did not differ between the ADHD and neurotypical groups (Fig. 1). Post hoc comparisons indicated that, relative to neurotypical adolescents, RXR-α levels were lower in both drug-naïve adolescents with ADHD (β = −0.411, SE = 0.126; exp[β] = 0.663; 95% CI 0.518–0.848; *p* = 0.001; Cohen’s d = 0.58) and adolescents with ADHD receiving medication (β = −0.468, SE = 0.124; exp[β] = 0.626; 95% CI 0.491–0.800; *p* < 0.001; Cohen’s d = 0.65) (Fig. 2). Finally, Table 2 showed that levels of RXR-α negatively associated with the mean reaction time in the go/no-go task (β = -0.001; Wald χ2 = 0.475; *p* = 0.029) after adjusting for group, age, sex, BMI, ADHD medications, SNAP-IV scores, and CBCL-DP scores, indicating that reduced RXR-α levels were associated with the longer mean reaction time in the go/no-go task.

**Table 2:** GLMs with adjustment of group, age, sex, BMI, ADHD medications, SNAP-4 scores, and CBCL-DP scores for associations between the levels of retinoid X receptor-α and cognitive function

	Errors	Mean reaction time (ms)	s.d. of reaction times (ms)
	Go/no-go task		
Levels of retinoid X receptor-α			
β	-0.005	-0.001	0.000
Wald χ2	0.030	4.750	0.070
p-value	0.863	**0.029**	0.791

	Working memory task		
Levels of retinoid X receptor-α			
β	-0.015	0.000	0.000
Wald χ2	3.024	0.904	0.002
p-value	0.082	0.342	0.963

ADHD: attention deficit hyperactivity disorder; BMI: body mass index; CBCL-DP: Childbehavior checklist dysregulation profile; GLM: generalized linear model; SNAP-4: the Swanson,Nolan, and Pelham Rating Scale-IV. *Note:*boldtype indicates statistical significance (p < 0.05).

## Discussion

Our findings partially supported the hypothesis that adolescents with ADHD have an impaired RXR–PPAR-γ axis. These individuals had lower levels of RXR-α, but not PPAR-γ or PPAR-γ co-A, than did their neurotypical counterparts, independent of ADHD medication use. Furthermore, lower RXR-α levels were associated with poorer inhibitory control function, as indicated by a longer mean reaction time in the go/no-go task.

RXR-α, a member of the nuclear receptor superfamily, exhibits high evolutionary conservation within its ligand-binding domain. Evidence suggests that RXR-α regulates unesterified docosahexaenoic acid–dependent synapse formation in cortical pyramidal neurons [17]. Both docosahexaenoic acid and cortical pyramidal neuron function are essential for brain development and cognitive performance and are implicated in the pathophysiology of ADHD [18, 19]. Spathis et al. reported that RXR-α signaling regulates the transcription of dopamine-biosynthesizing genes (e.g., tyrosine hydroxylase), potentially contributing to the neuroprotection of dopaminergic neurons [20]. A preclinical study demonstrated that IRX4204, a selective RXR-α agonist, dose-dependently enhanced the survival of tyrosine-hydroxylase-expressing dopaminergic neurons in the ventral midbrain [21]. These findings corroborate those of Li et al., who found that children with ADHD had lower levels of retinol—a natural RXR-α agonist—than did neurotypical children [7].

To the best of our knowledge, the present study is the first to identify an association between the RXR-α and ADHD, independent of medication status and symptom severity. Both drug-naïve and medicated adolescents with ADHD had significantly lower serum RXR-α levels than did neurotypical adolescents. Therefore, reduced RXR-α levels may play a crucial role in ADHD. Furthermore, we identified an association between reduced RXR-α levels and inhibitory control deficits in adolescents with ADHD. Wietrzych et al. assessed working memory function in mice with null mutations of RAR and found that the knockout mice exhibited significant working memory deficits [22]. The researchers suggested that impairment of RXR signaling in the frontal cortex led to these deficits [22]. The prefrontal cortex (PFC) plays a central role in executive functions and cognitive processes, such as working memory and inhibitory control. Dysfunction of the PFC is widely recognized as a core pathomechanism underlying ADHD [23]. Shibata et al. elucidated the key role of retinoic acid–RAR signaling in PFC development [24]. They found that genes regulated by retinoic acid are expressed in the neocortex of humans and macaques during the early to mid-stages of fetal development [24]. Mice lacking RXR exhibited molecular disorganization in the PFC and motor cortex as well as disrupted development of PFC–mediodorsal thalamic connectivity [24]. A functional magnetic resonance imaging study involving 12 individuals with ADHD revealed markedly reduced activation in the left PFC during a working memory task compared with the levels in neurotypical individuals [25]. These findings support our results, suggesting that reduced serum RXR-α levels impair PFC-related function, such as inhibitory control, in adolescents with ADHD.

This study has several limitations. First, to avoid exacerbation of attention and behavioral symptoms, 54 adolescents with ADHD (51.9%) were allowed to continue their prescribed medications during the cognitive assessments and blood analyses. However, the serum RXR-α levels did not differ between the medicated and drug-naïve ADHD groups. Further studies would be required to elucidate whether ADHD medications may affect the RXR–PPAR-γ axis. Second, only working memory and go/no-go tasks were conducted in our study. Associations of other cognitive functions (broader executive functions) with RXR–PPAR-γ axis factors require further investigation. Third, all RXR–PPAR-γ axis factor levels were only measured once. Further studies with the repeat measurement would be required to validate our findings. Fourth, after the multiple comparison correction (adjusted p-value = 0.05/3 = 0.0167), the association between reduced serum RXR-α levels and mean reaction time in the go/no-go task did not remain statistically significant. Further studies with a large sample size would be required to elucidate this association. Fifth, sex and BMI—covariates adjusted for in the GLMs—differed significantly between groups. We did not conduct additional sex- or obesity-stratified analyses because subgroup sample sizes were insufficient for adequately powered comparisons. Larger studies with adequate stratification are needed to investigate these effects. Sixth, to our knowledge, no study has directly compared RXR-α levels between cerebrospinal fluid (CSF) and serum. Whether peripheral RXR-α reflects CSF concentrations remains uncertain and warrants investigation. Paired CSF–serum studies comparing adolescents with ADHD and neurotypical controls are needed to address this gap. Seventh, we measured three RXR–PPAR-γ axis factors in the present study. After the multiple comparison correction (adjusted p-value = 0.05/3 = 0.0167), the between-group difference in the RXR-α levels remained significant. Finally, all our participants were Taiwanese adolescents, which may limit the generalizability of our findings to other ethnic and age groups.

## Conclusion

In this preliminary cross-sectional study comparing neurotypical adolescents with adolescents with ADHD, we observed lower RXR-α levels in the ADHD group, independent of current ADHD medication use. Exploratory analyses found that lower RXR-α levels were modestly associated with poorer inhibitory control, findings that should be considered hypothesis-generating and require confirmation in longitudinal and mechanistic studies. Additional studies are required to elucidate the role of the RXR–PPAR-γ axis, particularly RXR-α, in the neurobiological mechanisms underlying ADHD and related cognitive impairments.

## References

[1] Sayal K, Prasad V, Daley D, Ford T, Coghill D (2018) ADHD in children and young people: prevalence, care pathways, and service provision. Lancet Psychiatry 5:175–186 29033005 10.1016/S2215-0366(17)30167-0

[2] Franke B, Michelini G, Asherson P, Banaschewski T, Bilbow A, Buitelaar JK, Cormand B, Faraone SV, Ginsberg Y, Haavik J, Kuntsi J, Larsson H, Lesch KP, Ramos-Quiroga JA, Rethelyi JM, Ribases M, Reif A (2018) Live fast, die young? A review on the developmental trajectories of ADHD across the lifespan. Eur Neuropsychopharmacology: J Eur Coll Neuropsychopharmacol 28:1059–1088 10.1016/j.euroneuro.2018.08.001 PMC6379245 30195575

[3] Nierenberg AA, Ghaznavi SA, Sande Mathias I, Ellard KK, Janos JA, Sylvia LG (2018) Peroxisome Proliferator-Activated receptor gamma Coactivator-1 alpha as a novel target for bipolar disorder and other neuropsychiatric disorders. Biol Psychiatry 83:761–769 29502862 10.1016/j.biopsych.2017.12.014

[4] Nisbett KE, Pinna G (2018) Emerging therapeutic role of PPAR-alpha in cognition and emotions. Front Pharmacol 9:998 30356872 10.3389/fphar.2018.00998 PMC6190882

[5] Sharma S, Shen T, Chitranshi N, Gupta V, Basavarajappa D, Sarkar S, Mirzaei M, You Y, Krezel W, Graham SL, Gupta V (2022) Retinoid X receptor: cellular and biochemical roles of nuclear receptor with a focus on neuropathological involvement. Mol Neurobiol 59:2027–2050 35015251 10.1007/s12035-021-02709-y PMC9015987

[6] Qian L, Zhu Y, Deng C, Liang Z, Chen J, Chen Y, Wang X, Liu Y, Tian Y, Yang Y (2024) Peroxisome proliferator-activated receptor gamma coactivator-1 (PGC-1) family in physiological and pathophysiological process and diseases. Signal Transduct Target Ther 9:50 38424050 10.1038/s41392-024-01756-w PMC10904817

[7] Li HH, Yue XJ, Wang CX, Feng JY, Wang B, Jia FY (2020) Serum levels of vitamin A and vitamin D and their association with symptoms in children with attention deficit hyperactivity disorder. Front Psychiatry 11:599958 33329153 10.3389/fpsyt.2020.599958 PMC7719622

[8] Dumetz F, Bure C, Alfos S, Bonneu M, Richard E, Touyarot K, Marie A, Schmitter JM, Bosch-Bouju C, Pallet V (2020) Normalization of hippocampal retinoic acid level corrects age-related memory deficits in rats. Neurobiol Aging 85:1–10 31689598 10.1016/j.neurobiolaging.2019.09.016

[9] Locci A, Pinna G (2019) Stimulation of peroxisome Proliferator-Activated Receptor-alpha by N-Palmitoylethanolamine engages allopregnanolone biosynthesis to modulate emotional behavior. Biol Psychiatry 85:1036–1045 30955840 10.1016/j.biopsych.2019.02.006

[10] Wang J, Song HR, Guo MN, Ma SF, Yun Q, Zhang WN (2020) Adult conditional knockout of PGC-1alpha in GABAergic neurons causes exaggerated startle reactivity, impaired short-term habituation and hyperactivity. Brain Res Bull 157:128–139 32057952 10.1016/j.brainresbull.2020.02.005

[11] Akhondzadeh S, Mohammadi MR, Momeni F (2005) Passiflora incarnata in the teartment of attention-deficit hyperactivity disorder in children and adolescents. Therapy 2:609–614

[12] Chen MH, Pan TL, Lan WH, Hsu JW, Huang KL, Su TP, Li CT, Lin WC, Wei HT, Chen TJ, Bai YM (2017) Risk of suicide attempts among adolescents and young adults with autism spectrum disorder: A nationwide longitudinal Follow-Up study. J Clin Psychiatry 78:e1174–e1179 28872268 10.4088/JCP.16m11100

[13] Chen MH, Li CT, Lin WC, Hong CJ, Tu PC, Bai YM, Cheng CM, Su TP (2018) Cognitive function of patients with treatment-resistant depression after a single low dose of ketamine infusion. J Affect Disord 241:1–7 30081380 10.1016/j.jad.2018.07.033

[14] Bai YM, Liu YL, Kuo HW, Tsai SJ, Hsu JW, Huang KL, Tu PC, Chen MH (2023) Procollagen type 1 N-terminal propeptide, neurofilament light chain, Proinflammatory cytokines, and cognitive function in bipolar and major depressive disorders: an exploratory study of brain- bone axis and systemic inflammation. J Psychiatr Res 158:403–408 36657346 10.1016/j.jpsychires.2023.01.012

[15] Gau SS, Shang CY, Liu SK, Lin CH, Swanson JM, Liu YC, Tu CL (2008) Psychometric properties of the Chinese version of the Swanson, Nolan, and Pelham, version IV scale - parent form. Int J Methods Psychiatr Res 17:35–44 18286459 10.1002/mpr.237 PMC6878250

[16] Wu SJ, Hsu JW, Huang KL, Bai YM, Tu PC, Chen MH (2022) Functional dysconnectivity of cerebellum and attention networks in emotional dysregulation shared between attention deficit hyperactivity disorder and major depressive disorder: a multimodal imaging study. CNS Spectr 28:1–8 10.1017/S1092852922000876 35761511

[17] Cao H, Li MY, Li G, Li SJ, Wen B, Lu Y, Yu X (2020) Retinoid X receptor alpha regulates DHA-Dependent spinogenesis and functional synapse formation in vivo. Cell Rep 31:107649 32433958 10.1016/j.celrep.2020.107649

[18] Liu TH, Wu JY, Huang PY, Lai CC, Chang JP, Lin CH, Su KP (2023) Omega-3 polyunsaturated fatty acids for core symptoms of Attention-Deficit/Hyperactivity disorder: A Meta-Analysis of randomized controlled trials. J Clin Psychiatry 84:22r14772 10.4088/JCP.22r14772 37656283

[19] Cheng J, Liu A, Shi MY, Yan Z (2017) Disrupted glutamatergic transmission in prefrontal cortex contributes to behavioral abnormality in an animal model of ADHD. Neuropsychopharmacology 42:2096–2104 28176786 10.1038/npp.2017.30 PMC5561342

[20] Spathis AD, Asvos X, Ziavra D, Karampelas T, Topouzis S, Cournia Z, Qing X, Alexakos P, Smits LM, Dalla C, Rideout HJ, Schwamborn JC, Tamvakopoulos C, Fokas D, Vassilatis DK (2017) Nurr1:RXRalpha heterodimer activation as monotherapy for parkinson’s disease. Proc Natl Acad Sci U S A 114:3999–4004 28348207 10.1073/pnas.1616874114 PMC5393203

[21] Wang J, Bi W, Zhao W, Varghese M, Koch RJ, Walker RH, Chandraratna RA, Sanders ME, Janesick A, Blumberg B, Ward L, Ho L, Pasinetti GM (2016) Selective brain penetrable Nurr1 transactivator for treating parkinson’s disease. Oncotarget 7:7469–7479 26862735 10.18632/oncotarget.7191 PMC4884932

[22] Wietrzych M, Meziane H, Sutter A, Ghyselinck N, Chapman PF, Chambon P, Krezel W (2005) Working memory deficits in retinoid X receptor gamma-deficient mice. Learn Mem 12:318–326 15897255 10.1101/lm.89805 PMC1142461

[23] Arnsten AF (2009) Toward a new Understanding of attention-deficit hyperactivity disorder pathophysiology: an important role for prefrontal cortex dysfunction. CNS Drugs 23(Suppl 1):33–41 19621976 10.2165/00023210-200923000-00005

[24] Shibata M, Pattabiraman K, Lorente-Galdos B, Andrijevic D, Kim SK, Kaur N, Muchnik SK, Xing X, Santpere G, Sousa AMM, Sestan N (2021) Regulation of prefrontal patterning and connectivity by retinoic acid. Nature 598:483–488 34599305 10.1038/s41586-021-03953-x PMC9018119

[25] Wolf RC, Plichta MM, Sambataro F, Fallgatter AJ, Jacob C, Lesch KP, Herrmann MJ, Schonfeldt-Lecuona C, Connemann BJ, Gron G, Vasic N (2009) Regional brain activation changes and abnormal functional connectivity of the ventrolateral prefrontal cortex during working memory processing in adults with attention-deficit/hyperactivity disorder. Hum Brain Mapp 30:2252–2266 19107748 10.1002/hbm.20665 PMC6870879

